# Progress in Gynæcology

**Published:** 1901-09-21

**Authors:** 


### Progress in Gynæcology.

(*Continued from page* 397.)

**Cancer of the Uterus.**—J. Furneaux Jordan,[8] writing on this subject, refers to the recent investigations which support the view that the disease is due to some lowly organism, probably having its host in the vegetable kingdom; and he points out that although we are not quite certain as to the actual direct cause, we have now a very fair knowledge of many of the indirect causes, such as long continued irritation, as in chronic cervical catarrah, and the traumatism of childbirth. He lays stress on the extreme importance of vaginal examination in all cases of hæmorrhage or discharge, and points out that when these symptoms are accompanied with pain it usually means that the case has advanced beyond the possibility of radical treatment. He divides the treatment into palliative and radical measures. The former consists of antiseptic injections, curetting, and cauterisation, and the administration of opium; the latter of vaginal hysterectomy. Referring to the extensive operations that have been performed during the last few years by American and Continental operators, involving removal of the broad ligaments and their connective tissue together with the uterus by the combined abdominal and vaginal route, he points out that when recurrence takes place it nearly always occurs in the scar, and not in the pelvic lymphatic glands. Howard Kelly,[9] who was at one time a strong advocate of this method, has now come back to vaginal hysterectomy as the ideal treatment. He says that during the past year his views upon the extirpation of the cancerous uterus have undergone considerable change. He thinks that glandular metastases, which play such an important part in the extension of mammary cancer, are relatively unimportant, and, as a rule, only observed in the latest stages of uterine cancer. Histology shows that uterine cancer extends progressively from the cervix as a centre, therefore the chief aim of total hysterectomy by the vagina should be to give the diseased cervix the widest possible berth. He insists on the necessity of catheterisation of the ureters, advises that the vaginal mucous membrane should be incised at least 2 to 2½ c.m. distant from the disease, and does not hesitate to remove a portion of the bladder, or resect a ureter if necessary.

**Ectopic Gestation.**—The early diagnosis of extra-uterine pregnancy is often attended with considerable difficulty—many of the classical symptoms as described in most text-books being absent. E. H. Grandin[10] points out that in many cases there is no history of amenorrhœa, but on careful inquiry the patient admits that the last period was more scanty than usual; this, he thinks, is a point of considerable importance. Then again ectopic gestation might exist without associated uterine enlargement. He advocates that in doubtful cases an exploratory vaginal sec tion should be made, and quotes five cases to justify this statement. H. N. Vineberg[11] also calls attention to the fact that rupture of an ectopic pregnancy may take place without the patient having missed a period. He lays stress on the difficulty often met with in diagnosing between extrauterine pregnancy and ordinary abortion, and points out that the flow attending a ruptured tubal pregnancy is usually less profuse and more irregular than that which accompanies abortion. Some writers attach great importance to the "colicky" nature of the pains in extrauterine pregnancy, but as Vineberg says, the character of the pain is often very variable, sometimes not being a prominent symptom at all, its existence only being elicited by repeated questioning. Then, again, expulsion of a decidual cast is regarded by some as a sign of great value in ectopic gestation. But in many cases no membrane may be discharged, the decidua being cast off in shreds, or undergoing degeneration; and the microscopist, unless he should find chronic villi present, is unable to distinguish between the decidual cells of a uterine and tubal pregnancy. Vineberg insists on the necessity of regarding with suspicion every case of apparently early abortion, and if there is any doubt about the diagnosis, he advises that a complete examination under an anæsthetic should be made.

**Carcinoma of the Fallopian Tube.**—Although the Fallopian tube is but rarely attacked by malignant disease, there are a sufficient number of cases on record to prove beyond doubt the possibility of its occurrence. Carcinoma is the variety of disease most frequently met with, the growth having a marked tendency to assume a papillomatous character. Many authorities maintain that cancer is specially liable to attack tubes which have been subject to chronic salpingitis, and a very instructive case supporting this view has been recorded by E. R. Le Count.[12] The growth in this case involved the left Falopian tube. The patient, aged 47, married, had one child 21 years previously, and since then has had many miscarriages, being unable to carry a child beyond the third or fourth month of pregnancy. For two years previous to operation the patient had suffered from abdominal pain, worse at the menstrual period, and for one year a steadily-increasing abdominal swelling had been felt. The abdomen was opened, and two gallons of ascitic fluid escaped; the left tube was very much enlarged and thickened, and was excised close to the cornu of the uterus. The uterine end tapered abruptly, and the abdominal end was the seat of an exuberant cauliflower-like growth of new tissue which appeared to have burst forth from the tube. On making a section through the long axis of the tube, its centre was found to be occupied by a soft material of a grey colour, which filled the canal. The muscular coat was thin, and the external surface covered with unbroken serous membrane, except at the external end, where the serous covering was overrun with tissue which had grown out of the abdominal ostium. On microscopic examination the structure was found to be essentially the same in all portions. Set upon the muscular coats, which were thin, were many papillary growths, consisting of tenuous stalks of connective tissue covered with epithelium. The epithelium consisted of many strata, of which only the deeper layers were of the columnar type. The patient made a good recovery from the operation, but recurrence took place. The abdomen was reopened six months after, when the entire peritoneum was found studded with papillomatous growths of variable size. The large masses of growth were removed and recovery followed without any special event. The ascites returned gradually, so that five months after the second operation paracentesis was necessary, and this had to be repeated every two or three weeks until her death, which occurred 15 months later.

[8] Bir. Med. Rev., May, 1901. [9] Practitioner, April, 1901. [10] Med. Rec Jan. 5, 1901. [11] Lancet, June 8, 1901. [12] Bull. of John Hopkins Hospita March, 1901.

